# Changing Patterns of H6 Influenza Viruses in Hong Kong Poultry Markets

**DOI:** 10.1155/2011/702092

**Published:** 2010-10-18

**Authors:** Hiroichi Ozaki, Yi Guan, Malik Peiris, Robert Webster, Richard Webby

**Affiliations:** ^1^Laboratory of Veterinary Microbiology, Faculty of Agriculture, Tottori University, Tottori 680-8553, Japan; ^2^Department of Microbiology, The University of Hong Kong, Pokfulan, Hong Kong; ^3^Division of Virology, Department of Infectious Diseases, St. Jude Children's Research Hospital, Memphis, TN 38105, USA

## Abstract

Until 2001, H6N1 influenza viruses in the Hong Kong bird markets were represented by a single stable A/teal/Hong Kong/W312/97-like lineage. Beginning in 2001, despite a reduction in overall prevalence, an increase was observed in the number of H6 viruses isolated from chickens and other hosts. To assess any changes in H6 viruses, we characterized 18 H6 viruses isolated in the Hong Kong bird markets from 2001 to 2003. Experimental data showed that the 2003 H6 viruses had similar infectivity for chickens as did A/teal/HK/W312/97, and they were unable to transmit. Although all hemagglutinin genes were closely related to A/teal/HK/W312/97, 7 isolates were reassortant viruses containing similar gene segments of co-circulating H9N2 or H5N1 viruses. The receptor specificity was different from that of A/teal/Hong Kong/W312/97. Interestingly, similar observations have been documented in H9N2 viruses in Hong Kong. This evolution strongly suggests that some change in the ecology of influenza in the region selected for these changes. Taken together, these findings suggest that the H6 influenza viruses isolated in the Hong Kong markets are not well adapted to chickens and that the likely continued source of these viruses are other “minor” poultry species in which they are undergoing genetic and biologic evolution.

## 1. Introduction

The introduction and subsequent sustained global spread in the human population of influenza A viruses with a novel hemagglutinin (HA) subtype leads to an influenza pandemic. The novel influenza pandemics that occurred during the last century resulted in considerable mortality and morbidity. Genetic investigations revealed that these pandemic strains were partially or entirely derived from viruses of avian origin [1–4] and that most of them first appeared in southern China, a hypothetical influenza epicenter [5]. 

The avian H5N1 influenza virus that infected poultry and humans in Hong Kong in 1997 caused the death of 6 of 18 persons that were confirmed infected with this virus [6–8]. This virus was proposed to be a naturally occurring avian virus produced by the reassortment of H5N1 and H9N2 or H6N1 viruses [6, 7]. 

H9N2 influenza viruses have become panzootic during the last decade and have been isolated from different types of terrestrial poultry worldwide [9–11]. Two distinct lineages of H9N2 viruses, represented by the prototype A/duck/Hong Kong/Y280/97 (H9N2) (Dk/HK/Y280/97) and A/quail/Hong Kong/G1/97 (H9N2) (Qa/HK/G1/97) viruses, have become established in terrestrial poultry: Dk/HK/Y280/97-like viruses are found predominantly in chickens whereas Qa/HK/G1/97-like viruses are most often found in quail [10, 12]. The Qa/HK/G1/97-like viruses are thought to have been involved in the generation of the highly pathogenic H5N1 virus first isolated in 1997 [12]. H9N2 viruses of each lineage have been isolated from humans [13, 14], and the Dk/HK/Y280/97-like lineage also has been isolated from pigs in southern China [15]. 

During 1997, an H6N1 influenza A virus, A/teal/Hong Kong/W312/97 was isolated from a green-winged teal [16]. Subsequent characterization of the virus showed that seven of its eight gene segments were closely related to those of the H5N1 influenza viruses isolated from humans in 1997 [6–8, 17]. Later studies showed that A/teal/Hong Kong/W312/97-like viruses continued to circulate in the Hong Kong live-bird markets and that all H6N1 viruses isolated up until 2000 belonged to this lineage [18]. Similarly, the host range of these H6N1 viruses remained stable, with most isolates originating from quail and “minor" poultry species such as chukka, pheasant, and guinea fowl but rarely originating from chickens. Beginning in 2001, the host range appeared to be changing, with an increase in the number of H6N1 viruses isolated from chicken and silky chicken. To address whether this phenomenon was real and, if so, whether it was due to a change in the H6N1 viruses circulating in the markets, we investigated the antigenic, genetic, and biologic characteristics of various H6 influenza viruses isolated from the Hong Kong live-bird markets between 2001 and 2003. This paper describes the results of these studies and demonstrates an abrupt increase in the genetic diversity of H6 viruses in these markets.

## 2. Materials and Methods

### 2.1. Viruses

The viruses isolated in the Hong Kong region and the abbreviations used in this study are listed in Table 1. The viruses were collected from January 2001 through April 2003. Viruses present in fecal and cloacal samples from various bird species were grown in 10- to 11-day-old embryonated chicken eggs.

### 2.2. Antigenic Analysis

The H6 virus antigens were compared by using the hemagglutination inhibition (HI) test as previously described in [19]. All sera were pretreated with the receptor-destroying enzyme from *Vibrio cholerae* (Denka Seiken, Tokyo) to abolish interference by nonspecific serum inhibitors. The viruses, which came directly from infected allantoic fluid, were diluted to doses equivalent to four HA units for use in the tests.

### 2.3. RNA Extraction, RT-PCR, and DNA Sequencing

Viral RNA was extracted from infected allantoic fluid by using RNeasy kits (Qiagen, Valencia, CA) according to the manufacturer's instructions. Reverse transcription and PCR were performed under standard conditions by using primers specific for the various genes of influenza viruses [20]. PCR products were purified by using a gel extraction kit (Qiagen). Sequencing reactions were performed by the staff of the Hartwell Center for Bioinformatics and Biotechnology at St. Jude Children's Research Hospital. Template DNA was sequenced by using dRhodamine dye terminator cycle sequencing ready reaction kits with AmpliTaq DNA polymerase FS (Perkin-Elmer Applied Biosystems, Inc. [PE/ABI], Foster City, CA) and synthetic oligonucleotides. Samples were subjected to electrophoresis, detection, and analysis on PE/ABI model 3700 DNA sequencing instruments.

### 2.4. DNA Sequence Analysis

DNA sequences were compiled and edited by using the Lasergene sequence analysis software package (DNASTAR, Madison, WI). Multiple sequence alignments were made by using CLUSTAL W [21], and phylogenetic trees were generated by using the neighbor-joining algorithm in the TreeView version 1.6.6 software package (available at http://taxonomy.zoology.gla.ac.uk/rod/treeview.html). Nucleotide and amino acid sequences of other influenza viruses were obtained from the Influenza Sequence Database of the Los Alamos National Laboratory [22].

### 2.5. Hemagglutination Assays

The ability of the H6 viruses to hemagglutinate chicken, horse, and guinea pig erythrocytes was determined as previously described in [23]. Briefly, 32 HA units of virus (as determined by binding to chicken erythrocytes) were used in a hemagglutination assay with 0.5% (V/V) horse or guinea pig red blood cells. End titers were determined after one-hour incubation at room temperature. Chicken and guinea pig erythrocytes contain both *α*2-3- and *α*2-6-linked sialic acid whereas those from horse contain almost exclusively *α*2-3 linkages [24], thereby indirectly inferring receptor preferences of the agglutinating viruses.

### 2.6. Growth of Viruses in Quail and Chickens

We used 5-to 6-week-old Japanese quail and white leghorn chickens to determine the 50% infectious doses (QID_50_ and CID_50_) of a selection of H6 viruses. Infectious allantoic fluid 10-fold serially diluted (1 : 10 to 1 : 10^8^) was intranasally administered to three quails (0.2 ml inoculum) or chickens (0.5 ml inoculum) of each group. Birds were examined daily for disease signs, and cloacal and tracheal swabs were taken 3 and 5 days after inoculation. Tracheal swabs were placed in 0.5 ml of sample medium (50% glycerol in phosphate-buffered saline (PBS) that contained 1000 U/ml penicillin, 200 *μ*g/ml streptomycin, 50 U/ml mycostatin, 100 U/ml polymyxin B, and 250 *μ*g gentamicin); cloacal swabs were placed in 1 ml of sample medium. Each of two embryonated chicken eggs were inoculated with 100 *μ*l of the sample medium containing tracheal or cloacal swabs and were incubated for 48 hours at 37°C. HA assays using chicken red blood cells were performed to verify virus growth in the harvested allantoic fluid [19]. 

Transmission experiments were performed by following the method of Perez et al. [25]. Briefly, uninfected birds were placed in direct contact with or in cages above and below the inoculated birds (10^5^ pfu per bird) 1 day after inoculation. Trays between the cages were removed to allow efficient aerosol and fecal transmission. Animal work was performed under ABSL3+ biosafety conditions at St. Jude Children's Research Hospital. 

## 3. Results

### 3.1. H6 Influenza Viruses from Poultry in Hong Kong

Intensive surveillance systems in the Hong Kong live-bird markets have been in place since 1997. From 2001 through 2003, these systems showed that 253 isolates of 42495 tested specimens were H6 influenza viruses (232 in 2001, 12 in 2002, and 9 in 2003). We arbitrarily selected 18 H6 influenza viruses isolated from domestic poultry for further study (Table 1).

### 3.2. Antigenic Analysis

Since 1997, H5 viruses in Hong Kong have undergone antigenic and genetic change. To determine whether similar antigenic changes have occurred in H6 viruses in Hong Kong, we characterized the antigens of recent isolates by using HI tests. The H6 influenza viruses isolated in Hong Kong reacted with hyperimmune goat antisera raised against A/turkey/Massachusetts/1/65 (H6N2) (Ty/MA/1/65), hyperimmune rabbit antisera raised against A/shearwater/Australia/1/72 (H6N5) (Sh/Aus/1/72), and postinfection chicken antisera against A/teal/Hong Kong/W312/97 (H6N1) (Tl/HK/W312/97), A/quail/Hong Kong/YU1654/00 (H6N1) (Qa/HK/YU1654/00), A/quail/Hong Kong/YU39/01 (H6N1) (Qa/HK/YU39/01), and A/duck/Shantou/5540/01 (H6N2) (Dk/ST/5540/01) (Table 2). The antisera against Ty/MA/1/65 and Sh/Aus/1/72 were broadly reactive against all recent H6 viruses, as were antisera to Qa/HK/YU39/01 and Dk/ST/5540/01. All 2002 and 2003 isolates were antigenically indistinguishable from other viruses isolated during the same year whereas some heterogeneity existed among the 2001 isolates. Although there were detectable differences in reactivity patterns to antiserum against Qa/HK/YU39/01 between viruses of subsequent years, the H6 viruses of 2001, 2002, and 2003 represented a relatively antigenically homogeneous group of viruses. They had, however, changed in comparison to Tl/HK/W312/97, particularly in reactivity to antisera against Ty/MA/1/65 and Sh/Aus/1/72.

### 3.3. Genetic Analysis of the HA and NA Genes

**Figure 1:**
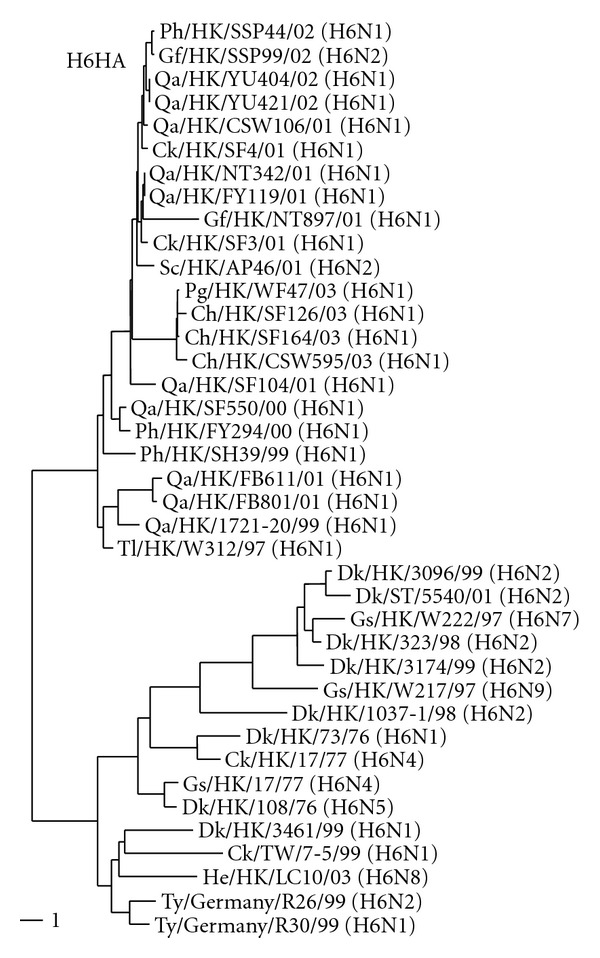
Phylogenetic analysis of the HA gene of Eurasian H6 influenza viruses.

The full-length HA sequences of the 18 H6 influenza viruses were determined to analyze their phylogenetic relationships (Figure 1). The HA gene of contemporary H6 influenza viruses isolated from terrestrial poultry had an open reading frame of 1704 bp that encodes a precursor protein of 567 amino acids. The insertion of a conserved aspartic acid between positions 144 and 145 (H3 numbering) of the precursor polypeptides distinguishes the contemporary terrestrial poultry viruses from aquatic bird viruses. All H6 isolates possessed the typical nonpathogenic sequence PQIETR/G at the HA cleavage site [26–28]. The homology between the HA nucleotide sequences of the contemporary isolates and that of Tl/HK/W312/97 ranged from 96% to 98%. The two strains isolated from chicken in 2001 (Ck/HK/SF3/01 and Ck/HK/SF4/01) were typical of other H6 viruses within the terrestrial host clade. 

In addition to the HA genes, the NA genes of all 18 H6 isolates were sequenced. The N1NA genes from 14 virus isolates from domestic poultry, including the two isolates from chicken, belonged to the cluster represented by Tl/HK/W312/97. This cluster was distinguishable from that containing A/goose/Guandong/1/96-like (H5N1) N1NA genes. The remaining two N1NA genes, which were from two of the 2001 isolates (Qa/HK/FB611/01 and Qa/HK/FB801/01), were slightly different from those of the Tl/HK/W312/97-like N1NA cluster and instead were more similar to that of A/quail/Hong Kong/1721-20/99. 

Alignment analysis revealed a 19-amino acid deletion from the stalk region of the N1NAs of all contemporary terrestrial isolates. This deletion is characteristic of the H6N1 viruses isolated in the region after 1997 [18]. Unexpectedly, two of the contemporary H6 viruses isolated from domestic poultry contained N2 genes. The N2NA gene of Sc/HK/AP46/01 was similar to that of Qa/HK/G1/97-like (H9N2) viruses (98% identity to A/HK/1074/99); the high degree of similarity suggests that Sc/HK/AP46/01 was a reassortant derived from cocirculating H6N1 and H9N2 viruses. The N2NA gene of Gf/HK/SSP99/02 did not belong to the Qa/HK/G1/97-like (H9N2) lineage but instead to a lineage represented by recent duck H9N2 isolates; this finding suggests reassortment between H6N1 viruses and aquatic bird viruses.

### 3.4. Genetic Analysis of Internal Genes

In 1998 and 1999, the H6N1 viruses isolated from the Hong Kong markets were genetically stable and were of the same genetic lineage [18]. To determine whether the stability of H6 viruses has continued since 1999, we analyzed partial sequences for each of the remaining genes from the isolated H6 viruses. Phylogenetic analysis of the PA polymerase (PA) gene revealed that, after 2001, reassortment occurred not only with NA genes but also with internal genes. PA genes from all nine viruses isolated in 2001 as well as those of Qa/HK/YU404/02, and Qa/HK/YU421/02 were similar to Tl/HK/W312/97-like viruses. The PA genes of Ph/HK/SSP44/02, Gf/HK/SSP99/02, and all of the 2003 isolates showed high homology to those of H5N1 viruses isolated from domestic poultry in 2000 and 2001. Also belonging to this lineage were the H5N1 viruses isolated from humans in Hong Kong in 2003 [29, 30]. The NP gene segments of Ph/HK/SSP44/02 and all of the 2003 isolates were distinct from that of the Tl/HK/W312/97-like lineage, as were the NS gene segments of Ph/HK/SSP44/02, Gf/HK/SSP99/02 and all of the 2003 isolates. These NP and NS gene segments were most similar to those of Dk/HK/Y280/97-like viruses (H9N2). The NP and NS gene segments of A/pheasant/HK/FY294/00 (H6N1) also clustered with those of Dk/HK/Y280/97-like viruses; this clustering indicated that the reassortment events between H6 and H9 viruses had happened as early as 2000. Although there were minor differences, the remaining gene segments (PB2, PB1, and M) of the H6 viruses isolated from domestic poultry belonged to the Tl/HK/W312/97-like lineage (Table 3).

### 3.5. DDBJ Accession Numbers

The nucleotide sequences for 18 H6 strains presented in this paper have been submitted to DNA Data Bank of Japan (DDBJ) under the accession numbers AB586744 to AB586887.

### 3.6. Hemagglutination Activity

The receptor specificity of the HA is considered a likely determinant of host range for influenza viruses. Additionally, studies have shown that H9N2 viruses circulating in the Hong Kong live-bird markets have human virus-like receptor specificity [31]. To explore the receptor binding specificity of Tl/HK/W312/97 and contemporary H6 isolates, the ability of these viruses to agglutinate erythrocytes from chicken, horse, and guinea pig was measured. Tl/HK/W312/97 and recent H6 viruses from aquatic birds [Dk/ST/5540/01 (H6N2) and He/HK/LC10/03 (H6N8)] were uniform in their ability to agglutinate erythrocytes from all sources to similar levels (Table 4). Conversely, the recent H6 isolates from domestic poultry were able to agglutinate erythrocytes of chicken and guinea pig origin but not those from horse. The binding of the contemporary H6 isolates was similar to that of Qa/HK/G1/97, a virus with human virus-like receptor specificity [31]. These results demonstrate that the receptor binding properties of the recent H6 isolates from poultry have evolved from those of Tl/HK/W312/97.

### 3.7. Experimental Infection of Quail and Chickens

An increase in the number of H6 viruses isolated from chickens in the Hong Kong live-bird markets raised the possibility that the host range of these viruses was expanding and that they were adapting to chickens. To test this hypothesis, we determined for chicken and quail the 50% infectious doses of selected H6 viruses isolated from different host species. Virus titers were standardized to 10^7.5^  to 10^8.0^  EID_50_/0.1 ml. Tl/HK/W312/97 and the domestic poultry H6 isolates from 2001, 2002, and 2003 were able to infect quail and chickens (Table 5), although in each case the ID_50_ was lower for quail than for chicken. None of the tested strains showed a markedly increased infectivity for chicken; the lack of an increase suggests that the contemporary H6 viruses, including those isolated from chickens, were not more adapted to chickens than was Tl/HK/W312/97.

### 3.8. Poultry-to-Poultry Transmission Experiment

 The ability of a virus to replicate within a host does not always correspond to its ability to be transmitted between individuals of that host. The ability of the domestic poultry H6 viruses to grow in chickens upon experimental infection conflicts with the low rates of isolation from this host in the markets. To determine the role of transmission in this apparent contradiction, we assessed the ability of the H6 viruses to be transmitted from chicken to chicken and from quail to chicken. Although the H6 viruses we selected replicated in the inoculated animals, no virus was isolated from the animals in direct contact with infected birds (Table 6); this result shows that transmission of the H6 viruses to chickens is poor. In contrast, Qa/HK/CSW106/01, Ck/HK/SF4/01, and Ph/HK/SSP44/02 were efficiently transmitted from infected to contact quail (data not shown). These infection and transmission results suggest that the H6 viruses circulating in southeastern China have not adapted to chicken populations and are unlikely to do so without further genetic change.

## 4. Discussion

The outbreak of H5N1 influenza in 1997 prompted researchers to hypothesize that chickens have the capacity to act as an intermediate host for influenza virus in humans. In light of this possibility, the observation that the host range of H6 viruses within the Hong Kong live-bird markets might be increasing to include chickens caused concern. H5N1 and H9N2 viruses that had been circulating in poultry in Hong Kong have caused human infection [7, 8, 13, 14, 17]. H6N1 viruses have been cocirculating with these H5N1 and H9N2 viruses in Hong Kong but have not been isolated from humans, and these viruses appear to have remained genetically stable with regard to antigenic drift and reassortment [18]. The results of our study show that, although the 2003 H6 viruses are not more adapted to chicken than are earlier isolates, H6 viruses in Hong Kong are evolving and reassorting with other viruses. 

Antigenic and genetic analyses of the H6 influenza viruses isolated from domestic poultry in southeastern China from 2001 through 2003 provide convincing evidence that the H6 influenza viruses are reassorting with H9N2, H5N1, and other viruses in the region. Previous studies have suggested that H9N2 viruses have a two-way transmission between terrestrial and aquatic birds [32]. This two-way transmission resulted in the generation of multiple genotypes of H9N2 viruses containing internal genes of aquatic avian origin. It is unclear whether the same type of transmission is occurring with H6N1 viruses or whether they are acquiring aquatic bird genes from H9N2 or similar viruses. Further interspecies transmission of these reassortants to other hosts may subsequently occur. This pattern parallels the re-emergence of H5N1 influenza viruses in Hong Kong in 2001 during which multiple new genotypes were identified [33]. Although all H6 HA genes appear to be of the Tl/HK/W312/97 lineage, the result of HI assays indicate that despite a high level of genetic similarity, there has been a gradual decline in the reactivity of the recent isolates to serum raised against isolates collected in 2001. 

 The ability of the Tl/HK/W312/97-like H6N1 or H6N2 viruses to replicate and transmit in quail but not in chickens suggested that the removal of quail from the Hong Kong markets (accomplished in February 2002) would substantially affect the isolation rates of H6 viruses [25]. In 2001, 232 H6 viruses were isolated in the market but only 12 during 2002 and 9 in 2003 were isolated. This dramatic reduction in the isolation rate does indeed suggest that quail were playing an important role in maintaining H6 viruses within the markets. The comparatively small number of birds such as chukkas and pheasants in the markets corresponds with the small number of H6 viruses isolated since the removal of quail. The lack of efficient transmission of the H6 viruses to chicken under experimental conditions, however, conflicts with the isolation of these viruses from chicken in the markets. One possible explanation for this is the nature of the surveillance sampling. Many market samples are fecal samples taken from cages beneath a given species of bird. Although the isolates are likely from the species housed in the above cage, there is the possibility of environmental contamination. A second possibility relates to the controlled nature of the experimental infections. It is likely that the birds in the markets are under additional stresses exerted by temperature and other environmental factors in addition to the presence of other pathogens. Such stresses which are impossible to mimic in laboratory settings may increase the susceptibility of the birds to influenza infection. Regardless, our results showing the unlikely role of chicken as efficient hosts of H6 viruses in the Hong Kong market system suggest that H6 viruses are now maintained by species such as chukkas and pheasants but not by chickens. Correspondingly, it should be expected that their incidence will remain low unless further adaptation to the chicken host is achieved.

In addition to the reduction in isolation rates, the increase in the genetic diversity within the H6 viruses in the markets also corresponds with the removal of quail. Exactly what role quail may have had in maintaining a stable Tl/HK/W312/97-like lineage is uncertain. Reassortants were present as early as 2000, but Tl/HK/W312/97-like viruses were the dominant genotype. It could be speculated that the Tl/HK/W312/97-like viruses are more adapted for replication and transmission in quail than some of the reassortant viruses. Hence, while quail remained in the market Tl/HK/W312/97-like viruses predominated. The association of Qa/HK/G1/97 (H9N2)-like viruses, that share 6 gene segments in common with Tl/HK/W312/97, with quail would support this possibility. It is of course also possible that it is coincidence that links the removal of quail and the emergence of multiple genotypes. Studies on H9N2 [34] and H5N1 [12] isolates in the region have also revealed an increasing degree of genetic diversity within these viruses. A more sinister scenario for the increase in H6 diversity is that there was an across-the-board enhancement in the amount of genetic reassortment occurring in the domestic poultry viruses throughout Southeast Asia.

 Although we were unable to detect an increase in the chicken infectivity of recent H6 viruses, some biologic differences to Tl/HK/W312/97 were seen. Similar to what has been described for H9N2 viruses [31], the contemporary H6 viruses have erythrocyte binding properties more reminiscent of human viruses. Human viruses, in contrast to avian viruses, are unable to bind to horse erythrocytes which contain primarily *α*2-3-linked sialic acids. Unlike the contemporary H6 viruses, Tl/HK/W312/97 has an avian virus-like binding pattern and retains the ability to bind to horse erythrocytes. Interestingly, the contemporary H6 viruses do not contain any of the amino acid changes in the HA that Matrosovich and colleagues associated with the human virus-like binding properties of the H9N2 viruses. Similarly, no correlation between predicted carbohydrate moieties and binding preferences of the H6 viruses was seen. It is interesting that both H9 and H6 viruses have evolved towards a human-like virus binding preference. The fact that it has occurred in distinct subtypes of influenza virus would strongly support a selective advantage accompaning the change. Exactly what environmental or host factor is driving the selective pressure is unclear. 

The hypothesis that the host range of H6 viruses underwent expansion in the Hong Kong markets appears to be incorrect in view of our findings. H6 viruses do have the capacity to be maintained in chicken populations as demonstrated by the emergence of multiple genotypes of H6N2 virus in chicken in the United States [35–37]. What further molecular changes are required for establishment of the Hong Kong H6 viruses in chickens, if at all possible, is unknown. Although the removal of quail has reduced the H6 virus burden in the Hong Kong bird markets, there has been an abrupt and corresponding increase in the genetic diversity of these viruses. It is unclear what effect this increase has on the continued presence and importance of these viruses for animal and human health.

## Figures and Tables

**Table 1:** H6 influenza viruses characterized in this study.

Group	Isolate^a^	Subtype	Abbreviation
2001 isolates	A/quail/HK/FB611/01	H6N1	Qa/HK/FB611/01
	A/quail/HK/FB801/01	H6N1	Qa/HK/FB/801/01
	A/quail/HK/SF104/01	H6N1	Qa/HK/SF104/01
	A/quail/HK/FY119/01	H6N1	Qa/HK/FY119/01
	A/quail/HK/NT342/01	H6N1	Qa/HK/NT342/01
	A/quail/HK/CSW106/01	H6N1	Qa/HK/CSW106/01
	A/guinea fowl/HK/NT897/01	H6N1	Gf/HK/NT897/01
	A/chicken/HK/SF3/01	H6N1	Ck/HK/SF3/01
	A/chicken/HK/SF4/01	H6N1	Ck/HK/SF4/01
	A/silky chicken/HK/AP46/01	H6N2	Sc/HK/AP46/01
2002 isolates	A/quail/HK/YU404/02	H6N1	Qa/HK/YU404/02
	A/quail/HK/YU421/02	H6N1	Qa/HK/YU421/02
	A/pheasant/HK/SSP44/02	H6N1	Ph/HK/SSP44/02
	A/guinea fowl/HK/SSP99/02	H6N2	Gf/HK/SSP99/02
2003 isolates	A/pigeon/HK/WF47/03	H6N1	Pg/HK/WF47/03
	A/chukkar/HK/SF126/03	H6N1	Ch/HK/SF126/03
	A/chukkar/HK/SF164/03	H6N1	Ch/HK/SF164/03
	A/chukkar/HK/CSW595/03	H6N1	Ch/HK/CSW595/03

^a^The letters in the strain identifiers relate to market of isolation, that is, all viruses with identifiers SSP were isolated from the same market.

**Table 2:** Antigenic analysis of H6 influenza viruses by hemagglutinin inhibition.

	Titer^a^ for					
Virus	Hyperimmune rabbit antisera against		Postinfection chicken antisera against			
	Ty/MA/1/65	Sh/Aus/1/72	Tl/HK/W312/97	Qa/HK/YU1564/00	Qa/HK/YU39/01	Dk/Shantou/5540/01
*Reference strains*						
Ty/MA/1/65	**2560**	5120	<	640	80	2560
Sh/Aus/1/72	80	**2560**	<	<	<	<
Tl/HK/W312/97	80	160	**2560**	2560	640	<
Qa/HK/YU1654/00	1280	<	320	**2560**	80	320
Qa/HK/YU39/01	160	640	40	320	**2560**	80
Dk/Shantou/5540/01	320	320	<	<	<	**1280**
*2001 isolates*						
Qa/HK/FB611/01	1280	2560	2560	2560	640	160
Qa/HK/FB/801/01	2560	5120	2560	5120	640	160
Qa/HK/SF104/01	5120	5120	2560	5120	5120	80
Qa/HK/FY119/01	1280	2560	640	2560	320	160
Qa/HK/NT342/01	1280	2560	640	2560	320	160
Qa/HK/CSW106/01	5120	5120	2560	5120	5120	80
Gf/HK/NT897/01	2560	5120	2560	5120	640	160
Ck/HK/SF3/01	5120	5120	640	5120	320	160
Ck/HK/SF4/01	5120	5120	640	5120	320	160
Sc/HK/AP46/01	2560	5120	1280	5120	320	160
*2002 isolates*						
Qa/HK/YU404/02	2560	2560	640	5120	640	160
Qa/HK/YU421/02	5120	5120	1280	5120	640	160
Ph/HK/SSP44/02	1280	5120	2560	2560	640	160
Gf/HK/SSP99/02	5120	5120	2560	5120	320	160
*2003 isolates*						
Pg/HK/WF47/03	2560	5120	640	5120	160	80
Ch/HK/SF126/03	2560	2560	640	2560	80	160
Ch/HK/SF164/03	2560	2560	640	2560	80	80
Ch/HK/CSW595/03	2560	2560	640	2560	80	80

Ty: turkey; MA: Massachusetts; Sh: shearwater; Aus: Australia; Tl: teal; HK: Hong Kong; Qa: quail; Ph: pheasant; Dk: duck; Gf: guinea fowl; Sc: silky chicken; Pg: pigeon. ^a^Titers in boldface are for homologous seta; <: no inhibition was detected at a serum dilution of 1 : 40.

**Table 3:** Genotyping of H6 subtype influenza viruses isolated in southeastern China (2001–2003).

	Gene segment^a^							
Virus	PB2	PB1	PA	HA	NP	NA	M	NS
Qa/HK/FB611/01	W	W	W	W	W	W	W	W
Qa/HK/FB/801/01	W	W	W	W	W	W	W	W
Qa/HK/SF104/01	W	W	W	W	W	W	W	W
Qa/HK/NT342/01	W	W	W	W	W	W	W	W
Qa/HK/NT342/01	W	W	W	W	W	W	W	W
Qa/HK/CSW106/01	W	W	W	W	W	W	W	W
Gf/HK/NT897/01	W	W	W	W	W	W	W	W
Ck/HK/SF3/01	W	W	W	W	W	W	W	W
Ck/HK/SF4/01	W	W	W	W	W	W	W	W
Sc/HK/AP46/01	W	W	W	W	W	G1	W	W
Qa/HK/YU404/02	W	W	W	W	W	W	W	W
Qa/HK/YU421/02	W	W	W	W	W	W	W	W
Ph/HK/SSP44/02	W	W	H5N1/01	W	Y280	W	W	Y280
Gf/HK/SSP99/02	W	W	H5N1/01	W	W	H9N2/00	W	Y280
Pg/HK/WF47/03	W	W	H5N1/01	W	Y280	W	W	Y280
Ch/HK/SF126/03	W	W	H5N1/01	W	Y280	W	W	Y280
Ch/HK/SF164/03	W	W	H5N1/01	W	Y280	W	W	Y280
Ch/HK/CSW595/03	W	W	H5N1/01	W	Y280	W	W	Y280

W: A/teal/Hong Kong/W312/97-like; G1: A/quail/Hong Kong/G1/97-like; Y280: A/quail/Hong Kong/Y280/97-like; in 2001 H9N2/00 similar to H9N2 isolated from ducks in 2000. ^a^Genotypes were established in light of the phylogenetic relationships.

**Table 4:** Hemagglutinating activity with erythrocytes from different animals.

	Hemagglutination with erythrocytes from		
	Chicken	Horse	Guinea pig
Tl/HK/W312/97 (H6N1)	32	32	16
Qa/HK/FB611/01 (H6N1)	32	<2	16
Qa/HK/FB/801/01 (H6N1)	32	<2	16
Qa/HK/SF104/01 (H6N1)	32	<2	16
Qa/HK/NT342/01 (H6N1)	32	<2	16
Qa/HK/FY119/01 (H6N1)	32	<2	16
Qa/HK/CSW106/01 (H6N1)	32	<2	32
Gf/HK/NT897/01 (H6N1)	32	<2	32
Ck/HK/SF3/01 (H6N1)	32	<2	16
Ck/HK/SF4/01 (H6N1)	32	<2	16
Sc/HK/AP46/01 (H6N2)	32	<2	16
Dk/ST/5540/01 (H6N2)	32	32	32
Qa/HK/YU404/02 (H6N1)	32	<2	16
Qa/HK/YU421/02 (H6N1)	32	<2	16
Ph/HK/SSP44/02 (H6N1)	32	<2	32
Gf/HK/SSP99/02 (H6N2)	32	<2	16
Pg/HK/WF47/03 (H6N1)	32	<2	16
Ch/HK/SF126/03 (H6N1)	32	<2	32
Ch/HK/SF164/03 (H6N1)	32	<2	16
Ch/HK/CSW595/03 (H6N1)	32	<2	16
He/HK/LC10/03 (H6N8)	32	32	16
Qa/HK/G1/97 (H9N2)	32	<2	16

**Table 5:** Experimental infection of quails and chickens.

Virus	QID_50_^a^	CID_50_^a^
Domestic poultry isolates		
2001 isolates		
Qa/HK/CSW106/01 (H6N1)	3.00	5.00
Ck/HK/SF4/01 (H6N1)	3.92	4.17
Sc/HK/AP46/01 (H6N2)	3.63	5.08
2002 isolates		
Ph/HK/SSP44/02 (H6N1)	2.20	4.58
Qa/HK/YU404/02 (H6N1)	2.50	4.75
Gf/HK/SSP99/02 (H6N2)	3.23	4.73
2003 isolates		
Ch/HK/CSW595/03 (H6N1)	2.73	4.73
Aquatic wild-bird isolates		
2001 isolate		
Dk/ST/5540/01 (H6N2)	2.6	6.48
2003 isolate		
He/HK/LC10/03 (H6N8)	3.17	7.92
Reference strain		
1997 isolate		
Tl/HK/W312/97 (H6N1)	3.47	4.92

^a^QID_50_ and CID_50_ indicate 50% quail and chicken infectious dose, respectively (log⁡_10_⁡EID_50_/0.1 ml). Each titer was obtained from tracheal swabs of animals at 3-day postinoculation.

**Table 6:** Experimental transmission of recent H6N1 viruses from quail to chicken and from chicken to chicken.

Virus	No. of positive trachea/total no. of birds [−log⁡_10_⁡(EID_50_/0.1 ml) for each bird]			
	Quails to chickens		Chickens to chickens	
	Inoculated^a^	Direct contact^b^	Inoculated^a^	Direct contact^b^
Qa/HK/CSW106/01	3/3(2.7, 2.0, 2.5)	0/3	3/3(3.0, 2.0, 2.3)	0/3
Ck/HK/SF4/01	3/3(2.0, 1.5, 2.7)	0/3	3/3(2.0, 1.5, 2.0)	0/3
Qa/HK/YU404/02	3/3(2.5, 2.5, 2.0)	0/3	3/3(1.0, 2.3, 2.5)	0/3
Ph/HK/SSP44/02	3/3(3.0, 2.7, 2.0)	0/3	3/3(2.3, 2.0, 2.7)	0/3
Ch/HK/CSW595/03	3/3(2.3, 2.0, 2.7)	0/3	3/3(2.3, 1.5, 2.5)	0/3

^a^Titers in parentheses correspond to each bird in the group 3 days after inoculation.

^b^Tracheal swabs were collected and titrated 5 days and 7 days after being put with inoculated group.
